# Modifiable drivers of community pharmacy closures in Colorado and Utah: a qualitative focus group study

**DOI:** 10.1093/haschl/qxag273

**Published:** 2026-10-09

**Authors:** Michael J DiStefano, Nai-Chia Chen, Carl V Asche, Kelly E Anderson, T Joseph Mattingly

**Affiliations:** Skaggs School of Pharmacy and Pharmaceutical Sciences, University of Colorado Anschutz Medical Campus, Aurora, CO 80045, United States; Center for Bioethics & Humanities, University of Colorado Anschutz Medical Campus, Aurora, CO 80045, United States; Skaggs School of Pharmacy and Pharmaceutical Sciences, University of Colorado Anschutz Medical Campus, Aurora, CO 80045, United States; College of Pharmacy, University of Utah, Salt Lake City, UT 84112, United States; Skaggs School of Pharmacy and Pharmaceutical Sciences, University of Colorado Anschutz Medical Campus, Aurora, CO 80045, United States; Program On Regulation, Therapeutics, And Law (PORTAL), Brigham and Women's Hospital and Har vard Medical School, Boston, MA, United States; College of Pharmacy, University of Utah, Salt Lake City, UT 84112, United States

**Keywords:** pharmacy closures, pharmacy access

## Introduction

The community pharmacy market in the United States is characterized by substantial churn and has seen a net decline in stores in recent years.^1^ Loss of access to community pharmacies has substantial impacts on patients, pharmacy staff and operations, and communities, including medication nonadherence, burnout, recruitment difficulties, and broader economic harm.^2^

Recent research examining the causes of closures has focused on the business practices of pharmacy benefit managers (PBMs).^3^ While PBM reform is gaining traction at both the state and federal level, identifying additional drivers of pharmacy closures could inform complementary policymaking and changes to practice aimed at curbing the pharmacy closures trend.

This study aimed to qualitatively describe the drivers of community pharmacy closures in Colorado and Utah from the perspectives of pharmacists and technicians who had experienced a recent pharmacy closure. In this research letter, we exclusively report findings related to drivers of pharmacy closures that are potentially modifiable through changes to policy or practice.

## Methods

We conducted 3 semistructured focus groups in September and October 2024 and analyzed the transcripts using a mixed deductive-inductive qualitative content analysis approach. The focus group guide and initial deductive themes and subthemes were informed by a conceptual framework grounded in the literature on pharmacy closures. This framework considers drivers of pharmacy closures at the community, pharmacy, and patient level. Additional details regarding recruitment, data collection, analysis, and limitations are included in the Supplemental Methods.

The Colorado Multiple Institution Review Board (COMIRB) determined this study to be exempt from review under Category 2, as it involves the conduction of interviews/focus groups (COMIRB #23-0928).

## Results

Thirteen participants were included across 3 focus groups (4-5 per group). Approximately half of the participants were female (7 of 13) and ages ranged from 20 to 73 years, with an average age of 37 years. Eight participants had experienced a pharmacy closure in Utah, and 5 had experienced a pharmacy closure in Colorado. Three participants owned pharmacies impacted by a closure, 4 worked as pharmacists at impacted pharmacies, and 6 worked as technicians. Two of the pharmacy closures occurred in rural towns (<1000 people), 5 were in small cities (50 000-100 000 people), 5 were in midsized cities (100 000-250 000 people), and 1 was in a large city (>250 000 people).

Table 1 provides illustrative focus group participant quotes related to modifiable drivers of pharmacy closures and links these drivers to potential policy and practice responses. Participants emphasized that community pharmacy viability is influenced by policy and regulatory environments that determine how easily pharmacies can obtain prescriptive authority and diversify revenue beyond prescription dispensing to include other pharmacist-provided clinical services. Adopting alternative revenue streams can also be more challenging in a rural setting. For example, unpredictable demand combined with cold-storage requirements means that administering vaccines is not always a reliable source of alternative revenue. Broader economic shifts can contribute substantially to pharmacy closures, especially when pharmacies must increasingly rely on retail sales to offset declining reimbursement. Participants also noted that policies impacting the workforce can affect pharmacy staffing and resilience. For example, a Colorado-based participant described the state's technician licensing requirement as not fit-for-purpose in the context of independent pharmacies. The growing difficulty of attracting talented pharmacists to small towns or rural areas also emerged as an important driver of pharmacy closures. One participant noted that the increasing levels of debt faced by recent PharmD graduates makes it less realistic to commit to life in a small town when there is “no guarantee of being able to be profitable” (focus group 1, participant 1). This participant also described the importance of having a “real tie to the community” when deciding where to work and when weathering economic challenges. One participant noted that a lack of adequate training in pharmacy business management may also contribute to operational challenges given the complexity of payer contracting and reimbursement within the fragmented US health care system. Finally, several participants directly attributed some pharmacy closures in rural areas to retirement or the unexpected death of a store's sole pharmacist. In both instances, it can be difficult to replace the pharmacist given the dearth of trained professionals willing to relocate on short notice. One participant specifically highlighted the lack of professional guidance to support pharmacists in developing succession plans for situations such as these.

**Table 1:** Verbatim quotes illustrating modifiable drivers of pharmacy closures identified by focus group participants and potential policy and practice responses.

Illustrative quotes	Potential policy or practice responses
**Community-level drivers**	
“I feel like with the prescriptive authority with pharmacists…the infrastructure that's set up for it…it's not optimal. We gotta go through a lot of paperwork. We gotta do all this stuff…And you know it's a pain. And then we have [that] on top of the current work. So a lot of pharmacists that I see don't really push for those extra opportunities to make a little bit better margin.” (Focus group 1, participant 2)	Reduce administrative frictions Expand pharmacist independent authority (vs collaborate practice agreements) to more clinical services One-stop/universal credentialing for commercial payer reimbursement
“…if you aren't able to offer, you know, clinical services, vaccines, ambulatory care, collaborative practice with providers…even something like annual wellness visits to, you know, really kind of think outside the box…if you're an independent pharmacy, you're not going to be very successful….And a lot of this stuff is going to be gated, too. Like you're going to need your accreditation. You're going to need special contracts with the pharmacy. You're going to have to go schmooze up a provider to be able to get a collaborative practice agreement….Because of the way the law is written, you know, you can't just go out and go change and do stuff as a pharmacist and get reimbursed for it. So…it's just a whole bunch of compounding factors that really are against you if you're a community pharmacy.” (Focus group 3, participant 3)	
“[The licensure requirement in Colorado] has made hiring harder…from an independent pharmacy perspective relative to chain [pharmacies]. It's frustrating because we're very specific on what we allow our techs to do. I know what that tech is capable of. I'm not going to ask her to perform any kind of calculations.…She is going to scan this bottle, scan this label, if the machine says they match, she's good to go, you know? I'm not asking her to make a whole lot of complex decisions. Now, this tech over here, she's been doing this for 25 years, and she's got this down, and I know good and well she's got it down, so I'll ask her to do more. That's the kind of decision making that happens when you work with your techs for, I mean, I'm not kidding when I say.…I've got a tech who's been at the pharmacy for 35 years. But when you're in a chain, obviously, it's just, who's the pharmacist of the day? Who's the tech of the day? They maybe have never met. They don't know what they're capable of. So those [licensure requirements] have to be in place for different settings. But it's a frustrating thing in the independent world.” (Focus group 2, participant 4)	Tiered technician licensure requirements Interstate technician licensure reciprocity agreements
“It is really tough when the kids now have so much debt, and they want to come to a small town and start a pharmacy, and with reimbursements the way they are, and no guarantee of being able to be profitable.” (Focus group 1, participant 1)	PharmD loan repayment programs tied to rural pharmacy service Expanded remote PharmD degree programs Funded apprenticeship pipelines for local residents to train as technicians
“They're just used to bigger towns…[with] stuff to do there. It's tough for kids who have lived in the Denver area to come over here to [a rural town] that closes at seven every night.” (Focus group 1, participant 1)	
“[My staff]…is either entirely people who grew up in this region…or married somebody who grew up in this region….That's the only way you get people to move out here…we're all very invested in serving our population, because those are our people.” (Focus group 2, participant 4)	
“I tried to sell the store to somebody, and he lasted about a year and a half and then closed the store….He and his wife wouldn't move here, you know. They're PharmD graduates, and they didn't want to come to a small town with what they considered inferior school systems.” (Focus group 1, participant 1)	
“I couldn't order ten vaccines of, like, Prevnar, you know, for $3500 or Covid vaccines, because I didn't know that I could [administer] them in a period of time….It's really tough…to even be vaccine capable out here in a small town, because you just don't know that you can inject them soon enough to get your money back.” (Focus group 1, participant 1)	Capitated or population-based payment models for pharmacies serving rural populations
“I think that the more that you lose on a script, the more you're depending on your retail side to make that up. And so if your demographic in your area isn't able to support your retail side, too, then the pharmacy itself, you know, isn't enough to support it.” (Focus group 1, participant 3)	
**Pharmacy-level drivers**	
“I wish that it was part of the pharmacy curriculum for us to take…just a basics in business [course], how to read a profit and loss, some of the information about how insurance companies actually work, how the contracts actually work, and some of the ins and outs, because the people I know who have opened independent pharmacies… we don't always have the business background that we need.” (Focus group 2, participant 3)	Include more advanced business management training in PharmD core curricula
“It's one of those things that you worry about when you're the owner, and all that responsibility lays on your shoulders, and I don't know what would happen if I were to pass away. I don't know what would happen to my store. I don't have a plan for succession written out with somebody who knows what they're doing.…But there's no guidance on what do you do when you're a sole owner at a hundred percent of a small business.” (Focus group 2, participant 3)	Succession-planning support offered by state boards or professional associations

## Discussion

Our study findings indicate several potential policy and practice interventions beyond PBM reform that could improve community pharmacy resilience. Policies to reduce the administrative frictions associated with adopting alternative revenue models—such as expanding pharmacist independent authority (vs collaborative practice agreements) to a larger set of clinical services or “one-stop” credentialing for commercial payer reimbursement in states where it is permitted—could improve uptake. Tiered pharmacy technician licensure requirements matched to task complexity or work setting and interstate technician licensure reciprocity agreements could help pharmacies, especially those in rural and border state areas, to hire and retain staff. PharmD loan repayment programs tied to rural pharmacy service commitments, expanded remote PharmD degree programs, and state grants to fund apprenticeship pipelines for local residents to train as technicians could bolster the pharmacy workforce in areas that struggle to attract or retain talent. Additionally, PharmD programs should consider incorporating more advanced business management training that emphasizes specific operational skills in addition to general behavioral skills.^4^ To minimize disruptions at independent pharmacies following from the unexpected death or retirement of a sole pharmacist, state boards of pharmacies or pharmacy associations could offer succession-planning support. Finally, a structural issue raised by participants concerns the viability of low-volume pharmacies operating under traditional fee-for-service (FFS) reimbursement models. In sparsely populated rural communities, pharmacies lack the total prescription volume and associated revenue required to offset fixed operating costs, particularly in the context of declining dispensing margins or increasing operating expenses. Expecting pharmacies to remain financially viable solely through prescription dispensing misaligns payment structure with function as a care provider to a local population. Rather than relying exclusively on FFS, capitated or population-based payments, such as models used in primary care or rural hospital stabilization programs,^5^ could better support pharmacies that assume responsibility for managing medication access for rural patient populations.

## Supplementary Material

qxag273_Supplementary_Data

## References

[1] Mattingly TJ 2nd, Sahu M, Anderson KE. Community pharmacy turnover and context of openings and closings by ownership type. JAMA Health Forum. 2025;6(8):e251988. 10.1001/jamahealthforum.2025.1988 PMC12317352 40748545

[2] DiStefano MJ, Chen NC, Asche CV, Anderson KE, Mattingly TJ 2nd. The crux of the community: a qualitative focus group study of the impact of community pharmacy closures in Colorado and Utah. J Am Pharm Assoc (2003). 2026;66(1):102940. 10.1016/j.japh.2025.102940 41072612

[3] Bacci JL, Oster N, Dunlap BS, Skillman SM, Frogner BK. Pharmacy benefit manager policy and pharmacy stakeholder perceptions of financial pressures and closures. Health Aff Sch. 2026;4(8):qxag186. 10.1093/haschl/qxag186 PMC13594649 42774826

[4] Morbitzer KA, Eckel SF, McClurg MR, McLaughlin JE. Key business of health care topics and strategies to implement within doctor of pharmacy curricula. Am J Pharm Educ. 2023;87(3):ajpe9096. 10.5688/ajpe9096 PMC10159534 36195335

[5] Pines JM, Vats S, Zocchi MS, Black B. Maryland's experiment with capitated payments for rural hospitals: large reductions in hospital-based care. Health Aff (Millwood). 2019;38(4):594–603. 10.1377/hlthaff.2018.05366 30933597

