# Supplementary material for: Modifiable drivers of community pharmacy closures in Colorado and Utah: a qualitative focus group study
Source: Health Aff Sch. 2026 Oct 9;4(10):qxag273. doi: 10.1093/haschl/qxag273 (PMC13689695; doi:10.1093/haschl/qxag273)
Supplement: Supplementary file 1 [file qxag273_supplementary_data.zip › Supplemental Material.docx]

**Table of Contents**

1. Methods Appendix
2. Focus Group Guide
3. Codebook
1. **Methods Appendix**

This article reports findings related to the causes of pharmacy closures that were identified in a focus group study designed to identify both causes and impacts of pharmacy closures. An article reporting the findings related to the impacts of pharmacy closures was previously published:

DiStefano MJ, Chen NC, Asche CV, Anderson KE, Mattingly TJ 2nd. "The crux of the community": A qualitative focus group study of the impact of community pharmacy closures in Colorado and Utah. J Am Pharm Assoc (2003). 2026 Jan-Feb;66(1):102940.

Below, we provide additional methodological details regarding this focus group study. Many of these details were described previously in the article cited above.

*Recruitment*

Study advertisements were emailed to the University of Colorado pharmacy preceptors list (1200+ pharmacists across the state), placed in Colorado Pharmacists Society news bulletins and member forum pages (800+ pharmacists and technicians across the state), and emailed to a state-provided list of 8000+ pharmacists and technicians active in Utah. Interested respondents completed a brief eligibility survey. Eligible respondents were required to have worked at a community pharmacy that either closed or was impacted by a nearby closure between 2018 and 2024. The eligibility survey also supported purposive selection across different pharmacy roles (e.g. owner, pharmacist, technician) and pharmacy locations (e.g. rural, small cities, large cities).

*Data Collection and Analysis*

The semi-structured focus group guide (see below) was informed by a conceptual framework based on a review of the pharmacy access and closure literature. Potential patient-level drivers of pharmacy closures included the prevalence of acute and chronic disease, prevalence of patients seen in outpatient versus inpatient settings, and patient insurance types. Pharmacy-level drivers included total prescription volume, pharmacy revenue, staffing considerations (pharmacist and technician hours), and staff turnover. Community-level drivers included the local economy and community population (total population and population demographics) changes.

References:

Traynor AP, Sorensen TD. Assessing risk for loss of rural pharmacy services in Minnesota. J Am Pharm Assoc (2003). 2005 Nov-Dec;45(6):684-93.

Traynor AP, Sorensen TD, Larson T. The main street pharmacy: becoming an endangered species. *Rural MN J*. 2007;2(1):83-100.

Guadamuz JS, Alexander GC, Zenk SN, Kanter GP, Wilder JR, Qato DM. Access to pharmacies and pharmacy services in New York City, Los Angeles, Chicago, and Houston, 2015-2020. J Am Pharm Assoc (2003). 2021 Nov-Dec;61(6):e32-e41.

Guadamuz JS, Wilder JR, Mouslim MC, Zenk SN, Alexander GC, Qato DM. Fewer Pharmacies In Black And Hispanic/Latino Neighborhoods Compared With White Or Diverse Neighborhoods, 2007-15. Health Aff (Millwood). 2021 May;40(5):802-811.

Focus groups were conducted virtually using Zoom in September/October 2024, lasted 90 minutes each, and were led by a male research team member with several years of experience working as a pharmacist in and managing community pharmacies (now currently an academic researcher with PharmD, PhD, and MBA training). At least two other research members also attended each focus group. Focus group transcripts were generated using Zoom’s AI transcription service.

Two research team members familiarized themselves with each focus group transcript and reviewed them for accuracy by cross-checking against video and time-stamped audio recordings and against notes taken by the research team during the focus groups. Beginning with one-third of each focus group transcript, the two researchers independently applied both deductive codes reflecting the conceptual framework and focus group guide and inductive (i.e. emergent) codes capturing new themes and subthemes. The researchers used a semantic (i.e. descriptive) approach while coding and were permitted to apply multiple codes to individual quotes to capture potential relationships between themes. Following the initial round of coding, the researchers met to discuss and resolve inconsistencies with a third researcher and to update the codebook. This process was then repeated with another third of the transcripts until all material had been analyzed. All analysis was conducted using MAXQDA.

*Limitations*

This study was not designed to reach thematic saturation. As such, the results should not be interpreted as offering either a complete account of or a diagnosis of the major reasons for community pharmacy closures. Additionally, the results may not be transferrable beyond Colorado and Utah. However, we purposively selected participants from a range of community sizes to increase the likelihood that study findings would be broadly salient.

2. **Focus Group Guide**

Topics for probing and follow-up questions are italicized.

Causes of pharmacy closures

1. Participants were first asked to introduce themselves and answer two ice-breaker questions:
   a. How many years have you been involved in pharmacy?
   b. What is the best part of your day when working at the pharmacy?
2. Based on your experience with a pharmacy closure, what led to the pharmacy closing?
   a. ***Patient-level*** *factors include, but are not limited to, items such as the prevalence of acute and chronic disease, patients seen in outpatient versus inpatient settings, and patient insurance types.*
   b. ***Pharmacy-level*** *factors include, but are not limited to, total prescription volume, pharmacy revenue, staffing (pharmacist and technician hours), and staff turnover.*
   c. ***Community-level*** *factors include, but are not limited to, the local economy, community population (total population and population demographics) changes, and pharmacy deserts.*
3. Based on your experience, what are the most important drivers of pharmacy closures?
4. Before we move onto the next set of questions, were there any other reasons not mentioned that you believe played a major role in the pharmacy closure that you had experience with?

Impacts of pharmacy closures

5. In your opinion, how has a pharmacy closure impacted the care a patient receives?
   a. *Did the closure impact patients’ relationships with the pharmacist, technicians, or other pharmacy staff?*
   b. *Did the closure impact patients’ medication adherence?*
   c. *Did the closure impact patients’ medication costs or other costs (e.g., travel distance)?*
   d. *Were there any continuity of care challenges following the closure?*
   e. *Did you have any medication safety concerns when transferring patients’ medications to a new pharmacy (or receiving transfers from a closing pharmacy)?*
   f. *Did you have any concerns about certain patients being more impacted than others?*
   g. *Have you seen any potential benefits to patients that have resulted from a pharmacy closure (e.g., perhaps a patient ended up receiving additional coordination services)?*
6. In your opinion, how has a pharmacy closure impacted nearby pharmacies?
   a. *Was the impact overall positive (e.g., increased business) to other pharmacies?*
7. In your opinion, how does a pharmacy closure impact prescription volume and overall workload for the pharmacies surrounding the closed pharmacy?
   a. *Do most patients just transfer to one pharmacy or do they end up transferring to multiple places?*
   b. *How long does it take the new (receiving) pharmacy to adjust to the workload changes?*
   c. *Does accommodating the new patients impact care for current/existing patients?*
8. In your opinion, how has a pharmacy closure impacted the technicians and pharmacists who worked for the pharmacy at the time of closure?
9. In your opinion, how has a pharmacy closure impacted the technicians and pharmacists who worked for a nearby pharmacy?
10. In your opinion, does a pharmacy closure have any impact on pharmacist or technician morale or job satisfaction?
11. In your opinion, how does a pharmacy closure impact the economy of the local community?
12. In your opinion, are there any types of communities that seem to be impacted more or less by pharmacy closures?
13. Is there anything you’d like to add that we didn’t cover?
3. **Codebook**

| *Hierarchical Themes* | *Frequency* |
| --- | --- |
| **Community-level drivers** |  |
| PharmD challenges | 2 |
| Policy | 17 |
| Commercial development | 1 |
| Competition | 7 |
| Buyout | 1 |
| Rural area | 14 |
| Community population | 4 |
| Local economy health | 6 |
| Inflation | 1 |
| **Pharmacy-level drivers** |  |
| Drug purchasing | 1 |
| Existing contracts | 4 |
| Mismanagement | 7 |
| Challenges with buying pharmacy | 4 |
| Illegal activity | 8 |
| Controlled substances | 4 |
| Staffing | 3 |
| Pharmacist-patient relationship | 1 |
| Turnover | 4 |
| Retirement | 2 |
| Death | 2 |
| Succession planning | 5 |
| Wages/salary | 8 |
| Staff capabilities | 9 |
| Burnout | 3 |
| Ties to the community | 3 |
| Desirability of location | 6 |
| School systems | 1 |
| Family considerations | 2 |
| Revenue | 8 |
| Reimbursement | 38 |
| Specialty | 1 |
| Prescriptive authority | 1 |
| Prescription length | 1 |
| Bureaucracy | 4 |
| Adjustments | 1 |
| Medicaid/Medicare | 5 |
| 340B | 3 |
| Vaccines | 5 |
| Non-prescription drug revenue | 12 |
| Prescription volume | 1 |
| **Patient-level drivers** |  |
| Insurance difficulties | 2 |
| Convenience for patients | 3 |
| Patient mix | 4 |
